# Children with recurrent pneumonia and non-cystic fibrosis bronchiectasis

**DOI:** 10.1186/s13052-016-0225-z

**Published:** 2016-02-09

**Authors:** Maria Francesca Patria, Benedetta Longhi, Mara Lelii, Claudia Tagliabue, Marinella Lavelli, Carlotta Galeone, Nicola Principi, Susanna Esposito

**Affiliations:** Pediatric Highly Intensive Care Unit, Department of Pathophysiology and Transplantation, Università degli Studi di Milano, Fondazione IRCCS Ca’ Granda Ospedale Maggiore Policlinico, Via Commenda 9, 20122 Milan, Italy; Department of Clinical Sciences and Community Health, Università degli Studi di Milano, Via Commenda 9, 20122 Milan, Italy

**Keywords:** Bronchiectasis, Lower respiratory tract infections, Non-cystic fibrosis bronchiectasis, Pediatric pulmonology, Pneumonia, Recurrent pneumonia

## Abstract

**Background:**

Recurrent pneumonia (RP) is one of the most frequent causes of pediatric non-cystic fibrosis (CF) bronchiectasis (BE) and a consequent accelerated decline in lung function. The aim of this study was to analyse the clinical records of children with RP in attempt to identify factors that may lead to an early suspicion of non-CF BE.

**Methods:**

We recorded the demographic and clinical data, and lung function test results of children without CF attending our outpatient RP clinic between January 2009 to December 2013 who had undergone chest high-resolution computed tomography ≥8 weeks after an acute pneumonia episode and ≤6 months before enrolment.

**Results:**

The study involved 42 patients with RP: 21 with and 21 without non-CF BE. The most frequent underlying diseases in both groups were chronic rhinosinusitis with post-nasal drip and recurrent wheezing (81 % and 71.4 % of those with, and 85.7 % and 71.4 % of those without BE). FEV_1_ and FEF_25–75_ values were significantly lower in the children with non-CF BE than in those without (77.9 ± 17.8 *vs* 96.8 ± 12.4, *p* = 0.004; 69.3 ± 25.6 *vs* 89.3 ± 21.9, *p* = 0.048). Bronchodilator responsiveness was observed in seven children with BE (33.3 %) and two without (9.5 %; *p* = 0.13).

**Conclusions:**

Reduced FEV_1_ and FEF_25–75_ values seem associated with an increased risk of developing non-CF BE in children with RP. This suggests a need for further studies to confirm the diagnostic usefulness use of spirometry in such cases.

## Background

The development of non-cystic fibrosis bronchiectasis (non-CF BE), a chronic, progressive pulmonary disorder characterised by the permanent dilatation of one or more bronchi due to structural modifications in the bronchial wall [1], is thought to be mainly due to chronic inflammation of the lower respiratory tract. It is frequently diagnosed in children living in developing countries and those belonging to indigenous populations, including Australian aborigines, New Zealand Maoris and Alaskan natives [2], but it is less frequent in industrialised countries, probably because of differences in genetic and environmental predisposing factors, better hygiene and nutrition, greater vaccine coverage, and the availability of effective antimicrobial drugs. However, data collected in the UK [3, 4] and Italy [5] indicate that it is not rare, although its prevalence is probably underestimated mainly because it is only recently that the advent of high-resolution computed tomography (HRCT) has made it possible to identify cases that could not be diagnosed by means of bronchograms [6].

In pediatric patients, non-CF BE causes an accelerated decline in lung function that leads to repeated hospital admissions due to acute infectious exacerbations, a poorer quality of life, and possible premature death in early adult life [7]. The early identification of children with non-CF BE could therefore significantly improve their long-term prognosis by delaying or preventing progressive lung function abnormalities. Kapur et al. retrospectively reviewed the anthropometric and lung function data of 52 children with non-CF BE aged ≥3 years, and found that they remained stable for 3–5 years after the start of appropriate therapy [8]; however, exacerbations accelerated a decline in lung function, thus suggesting that every effort should be paid in order to ensure the early diagnosis of BE in children with repeated or chronic respiratory problems.

Recurrent pneumonia (RP), which occurs in 6.7-8.2 % of the general pediatric population in industrialised countries, is frequently described in association with a severe underlying disease favouring lung infections and is considered a marker of the possible development of non-CF BE [9–11]. However, it is often encountered in children without significant risk factors [12], and this can delay the diagnosis of non-CF BE itself, thus increasing the risk of worsening lung function during adolescence and adult life.

The aim of this study was to analyse the clinical records of a group of children with RP in detail in an attempt to identify factors that may lead to an early suspicion of non-CF BE, so leading to prompt diagnosis and implementation of the best prophylactic and therapeutic measures.

## Methods

### Study design

This was a retrospective cohort study that involved children regularly followed-up between January 2009 and December 2013 in the Respiratory Disease Section of the University of Milan (Fondazione IRCCS Ca’ Granda Ospedale Maggiore Policlinico, Milan, Italy) because of RP. In accordance with Wald [13], RP was defined as at least two episodes of radiographically confirmed alveolar pneumonia in one year or more than three episodes at any time, with radiographic clearance between episodes. The alveolar pneumonia diagnoses were all confirmed by chest radiographs evaluated by an independent expert radiologist who classified the findings in accordance with the World Health Organization criteria for the standardized interpretation of pediatric chest radiographs for the diagnosis of pneumonia [14]. According to a protocol active in the Unit during the study period, 8–12 weeks after the second episode of pneumonia all the children with RP performed a chest radiograph and those who did not show any evidence of pneumonia were eligible for the study. Further criteria for selection were: 1) absence of CF on the basis of a negative sweat test and genetic analysis, 2) availability of the results of a chest thoracic high-resolution computed tomography (HRCT) performed ≥8 weeks after the last acute pneumonia episode leading to RP diagnosis in order to avoid the risk of diagnosing transient bronchial dilation and ≤6 months before enrolment, 3) availability of all the demographic and clinical data of each patient, including those relating to evaluations of the possible cause of RP such as immune disorders (including atopy and allergy); upper airway problems such as chronic rhinosinusitis with post-nasal drip, recurrent wheezing, tuberculosis, middle lobe syndrome, congenital heart defects, and primary ciliary dyskinesia (PCD) and 4) availability of the results of the first pulmonary function test.

### Ethics, consent and permissions

The study was approved by the Ethics Committee of Fondazione IRCCS Ca’ Granda Ospedale Maggiore Policlinico, Milan, Italy, and written informed consent was obtained from the parent (s) or legal guardian (s) of each study participant, and from the children aged >8 years.

### Procedures

A record was made of all of the available demographic and clinical data of each patient. Regarding underlying predisposing diseases, all the studied conditions were considered only when a doctor diagnosed and documented their presence by radiological examination (i.e., chronic rhinosinusitis, middle lobe syndrome, heart disease) and/or laboratory test (i.e., allergy, immunodeficiencies). Chronic rhinosinusitis was defined as the persistence of clinical symptoms of rhinosinusitis for more than 90 days [15] and its diagnosis was confirmed at the time of the HRCT. Middle lobe syndrome was initially suspected because of chronic cough, RP and chest X-ray evaluation showing atelectasis of the right middle lobe and definitively confirmed by HRCT.

The HRCT was performed in any case following standard procedures. Inspiratory scans were from lung apices to diaphragm with a scan thickness of 1 mm, and a scan interval of 10 mm, with additional slices at 5-mm intervals for areas of particular concern. Expiratory scans were at intervals of 2–3 cm. A scan time of 1 sec was used. Image windowing and levels were standard, at W-1,500, L-600. Scans were reconstructed with a high-frequency (bone) algorithm. Tube parameters were KVp 120, and mAs 50–250. The smallest field of view that included the entire width of the chest at the diaphragm on the inspiratory scout view was used. Scans were independently evaluated by a consultant pediatric radiologist blinded to the cases HRCT scans were scored using a modification of the Bhalla score [16]. The scoring system was as follows: 1) extent of bronchiectasis (0 = none, 1 = one or partial broncho pulmonary segment involved, 2 = two or more bronchopulmonary segments involved, 3 = generalized cystic bronchiectasis); 2) severity of bronchial dilatation (0 = normal, 1 = less than twice the diameter of the adjacent pulmonary artery, 2 = more than twice the diameter of adjacent pulmonary artery); 3) severity of bronchial wall thickening (0 = normal, 1 = 0.5 × the diameter of the adjacent pulmonary artery, 2 = 0.5-1.0 × the diameter of the adjacent pulmonary artery, 3 = ≥ 1.0 × the diameter of the adjacent pulmonary artery); 4) presence of mucous plugging in large airways (0 = none, 1 = present); 5) presence of mucous plugging in small airways (0 = none, 1 = present); and 6) extent of decreased attenuation (0 = normal, 1 = ≤ 50 % of lobar volume, 2= > 50 % of lobar volume). Scores ≤5 were considered indicative of mild disease whereas scores between 6 and 10 and > 10 of moderate and severe disease, respectively.

According to the protocol active in the Unit, the first pulmonary function test was obtained from all of the clinically stable patients not receiving drugs that could influence test results in the same day during which HRCT was done. A HypAir Compaq spirometer (Medisoft, Belgium) on the basis of the American Thoracic Society/European Respiratory Society criteria was used [17]. The best expected values of at least three reproducible manoeuvres were considered, and forced vital capacity (FVC), forced expiratory volume in 1 second (FEV_1_), and forced expiratory flow 25-75 % (FEF_25–75_) were recorded. After the baseline evaluation, the patients were administered inhaled salbutamol (four 100 μg doses) by means of a metered dose inhaler. A positive bronchodilator response was defined as an improvement in FEV_1_ of at least 12 % (or at least 200 mL) in comparison with baseline. The results were expressed as percentages of the predicted value using Zapletal’s reference equation.

### Statistical analysis

The continuous data were expressed as mean values ± standard deviation [SD], and compared between groups using a Wilcoxon rank-sum test after checking that the data were normally distributed. The categorical data were expressed as number and percentages and compared using contingency table analysis and χ^2^ or Fisher’s exact test, as appropriate. Odds ratios (OR) and 95 % confidence intervals (CI) were calculated through unconditional multiple logistic regression models, adjusting for age at diagnosis (using a continuous term). All of the tests were two sided, and a p value of <0.05 was considered statistically significant. The data were analysed using SAS version 9.1 software (Cary, NC, USA).

## Results

Among the 435 patients regularly followed-up for RP, a total of 42 were selected for the study. A total of 393 cases were excluded, in 358 cases because of CF and in the remaining 35 because HRCT was done beyond the established period of time. None of them had a history of wet cough on a daily or regular basis at the time of the HRCT. Table 1 shows the demographic characteristics of the 42 enrolled patients with a history of RP: 21 had non-CF BE (14 males; mean age ± SD, 12.2 ± 4.5 years) and 21 normal HRCT findings (13 males; mean age ± SD, 9.2 ± 3.9 years; *p* = 0.02). Mean age ± SD at the time of the HRCT scan was significantly higher in the children with BE (6.6 ± 2.9 *vs* 4.7 ± 3.0 years; *p* = 0.03), but gestational age, birth weight, the number of patients with respiratory problems at birth, the number of patients exclusively breastfed for >3 months, the number of siblings, age at the beginning of day care attendance, and exposure to passive smoking were similar between the two groups. For the latter characteristics, no significant difference between groups emerged even after adjusting for age at diagnosis.

**Table 1:** Demographic characteristics of children with a history of recurrent pneumonia (RP), with and without non-cystic fibrosis bronchiectasis (non-CF BE)

	RP and non-CF BE (*n* = 21)	RP alone (*n* = 21)	*P*-value*	OR (95 % CI)**
Gender, F:M	7:14	8:13		
Mean age ± SD at enrolment, years	12.2 ± 4.5	9.2 ± 3.9	**0.02**	
Mean age ± SD at diagnosis, years	6.6 ± 2.9	4.7 ± 3.0	**0.03**	
Gestational age (weeks)				
<32	1 (4.7 %)	4 (19.0 %)		0.33 (0.03-3.65)
32-36	2 (9.5 %)	2 (9.5 %)		1.05 (0.12-8.95)
≥37	18 (85.7 %)	15 (71.4 %)	0.35	1 (ref)
Birth weight (grams)				
<2500	2 (10.0 %)	7 (33.3 %)		1 (ref)
≥2500	19 (90.5 %)	14 (66.7)	0.13	3.19 (0.53-19.2)
Respiratory distress at birth^1^				
No	16 (80.0 %)	13 (61.9 %)		1 (ref)
Yes	4 (20.0 %)	8 (38.1 %)	0.20	0.54 (0.12-2.32)
Intubation at birth^a^				
No	16 (84.2 %)	16 (76.2 %)		1 (ref)
Yes	3 (15.8 %)	5 (23.8 %)	0.70	0.79 (0.15-4.14)
Nasal CPAP at birth^a^				
No	17 (89.5 %)	16 (76.2 %)		1 (ref)
Yes	2 (10.5 %)	5 (23.8 %)	0.41	0.57 (0.09-3.72)
Oxygen therapy at birth^a^				
No	16 (84.2 %)	15 (71.4 %)		1 (ref)
Yes	3 (15.8 %)	6 (28.6 %)	0.46	0.64 (0.13-3.26)
Breastfeeding >3 months^a^				
No	8 (40.0 %)	12 (57.1 %)		1 (ref)
Yes	12 (60.0 %)	9 (42.9 %)	0.27	1.94 (0.52-7.28)
No. of siblings				
0	12 (57.1 %)	9 (42.9 %)		1 (ref)
1+	9 (42.9 %)	12 (57.1 %)	0.35	0.52 (0.14-1.91)
Age at start of day care attendance^a^				
<3 years	6 (31.6 %)	10 (52.6 %)		1 (ref)
≥3 years	13 (68.4 %)	9 (47.4 %)	0.19	2.06 (0.53-8.10)
Passive smoking exposure				
No	15 (71.4 %)	10 (47.6 %)		1 (ref)
Yes	6 (28.6 %)	11 (52.4 %)	0.12	0.28 (0.07-1.16)

*OR* odds ratio, *CI* confidence interval, *SD* standard deviation, *CPAP* continuous positive airway pressure. ^a^Some missing data. *χ^2^ or Fisher’s exact test (categorical variables); Wilcoxon’s rank-sum test (continuous variables). Significant differences are reported in bold. **ORs from logistic regression models, adjusting for age at diagnosis

Table 2 shows the underlying diseases in the study population. More than 90 % of the children enrolled in the study had an underlying disease without any statistically significant difference between the patients with and without non-CF BE. The most frequently reported diseases were chronic rhinosinusitis with post-nasal drip (17/21 children with BE, 81 %, and 15/21 without BE, 71.4 %) and recurrent wheezing (in 15/21 children with BE, 71.4 %, and 18/21 without BE, 85.7 %). IgA deficiency was diagnosed in 6 patients (3/21 children with BE, 14.3 %, and 3/21 without BE, 14.3 %), whereas primary ciliary dyskinesia was diagnosed in one child only with BE (1/21, 4.8 %). No cases of tuberculosis infection, foreign body retention or airway malformation were detected in either group.

**Table 2:** Underlying predisposing diseases in children with a history of recurrent pneumonia (RP), with and without non-cystic fibrosis bronchiectasis (non-CF BE)

	RP and non-CF BE (*n* = 21)	RP alone (*n* = 21)	*P*-value*	OR (95 % CI)**
Chronic rhinosinusitis with post-nasal drip				
No	4 (19.0 %)	6 (28.6 %)		1 (ref)
Yes	17 (81.0 %)	15 (71.4 %)	0.47	1.40 (0.30-6.45)
Atopy				
No	8 (38.1 %)	9 (42.9 %)		1 (ref)
Yes	13 (61.9 %)	12 (57.1 %)	0.75	0.79 (0.20-3.21)
Total IgE at diagnosis, KU/L	291.8 ± 363.1	278.5 ± 20.9	0.60	
Sensitisation to perennial allergens				
No	14 (66.7 %)	17 (81.0 %)		1 (ref)
Yes	7 (33.3 %)	4 (19.0 %)	0.29	1.81 (0.41-7.93)
Wheezing				
No	6 (28.6 %)	3 (14.3 %)		1 (ref)
Yes	15 (71.4 %)	18 (85.7 %)	0.45	0.31 (0.06-1.69)
IgA deficiency				
No	18 (85.7 %)	18 (85.7 %)		1 (ref)
Yes	3 (14.3 %)	3 (14.3 %)	1	0.83 (0.14-5.09)
Primary ciliary dyskinesia				
No	20 (95.2 %)	21 (100 %)		
Yes	1 (4.8 %)	0	1	NC
Middle lobe syndrome				
No	14 (66.7 %)	11 (52.4 %)		1 (ref)
Yes	7 (33.3 %)	10 (47.6 %)	0.35	0.66 (0.18-2.45)
Heart disease				
No	18 (85.7 %)	18 (85.7 %)		1 (ref)
Yes	3 (14.3 %)	3 (14.3 %)	1	1.71 (0.26-11.2)
Tuberculosis infection				
No	21 (100.0 %)	21 (100.0 %)		
Yes	0	0	1	NC
Foreign body retention				
No	21 (100.0 %)	21 (100.0 %)		
Yes	0	0	1	NC

*OR* odds ratio, *CI* confidence interval, *NC* not computable

*χ^2^ or Fisher’s exact test (categorical variables); Wilcoxon’s rank-sum test (continuous variables). **ORs from logistic regression models, adjusting for age at diagnosis

Among the children with non-CF BE, the HRCT abnormalities were focal in 14 (66.6 %) and multifocal in seven (33.4 %; unilateral in two and bilateral in five). In most of the cases (18, 85.7 %) at least one abnormality was evidenced in the same anatomical areas as the initial pneumonia. The right side was slightly more frequently involved than the left (56 % *vs* 44 %); the middle lobe was involved in 32.3 %, the right and left lower lobes in 17.6 % each, the lingula in 11.7 %, the left upper lobe in 8.8 %, and the right upper lobe and left hilum in 5.9 % each. The BE pattern was cylindrical in 18/21 patients (85.7 %) and varicose in three (14.3 %). The presenting symptoms at the time of HRCT scan were chronic productive cough in 18/21 (85.7 %), crepitations in the same lung area in four (19 %) and dyspnea in three (14.3 %). None of the patients showed clubbing or hemoptysis. According to HRCT scores, severity of bronchiectasis was considered mild in eleven (52.4 %) cases, moderate in seven (33.3 %), and severe in three (14.3 %).

Table 3 summarises the patients’ clinical history of lower respiratory tract involvement and the results of their lung function tests. All the children were entirely well between pneumonia episodes and showed an increase in inflammatory biomarkers (white blood cell count, neutrophils and C reactive protein) during the pneumonia. Age at the time of the first pneumonia episode, the number of pneumonia episodes experienced before undergoing HRCT, and the number of wheezing events per year were similar in those with and without non-CF BE, but pulmonary tests showed that the values of FEV_1_ and FEF_25–75_ were significantly lower in the children with non-CF BE (*p* = 0.004 and *p* = 0.048, respectively). After adjustment for age, the results were still statistically significant. Furthermore, seven of the children with BE (33.3 %) were responsive to bronchodilators as against only two of those without (9.5 %; *p* = 0.13).

**Table 3:** Clinical history of lower respiratory tract involvement and lung function test results in children with a history of recurrent pneumonia (RP), with and without non-cystic fibrosis bronchiectasis (non-CF BE)

	RP and non-CF BE	RP alone	*P*-value*	OR (95 % CI)**
Mean age ± SD at first pneumonia episode, years	2.0 ± 1.2	1.8 ± 1.6	0.35	1.07 (0.51-2.23)
Mean number of pneumonia episodes before HRCT ± SD	4.0 ± 3.2	4.1 ± 2.3	0.30	0.65 (0.34-1.25)
Mean number of wheezing events per year ± SD	4.5 ± 2.9	5.0 ± 3.3	0.60	0.90 (0.45-1.81)
First available lung function test results:				
FVC (% of predicted value)	91.1 ± 15.8	100.5 ± 10.6	0.13	0.52 (0.24-1.13)
FEV_1_ (% of predicted value)	77.9 ± 17.8	96.8 ± 12.4	**0.004**	**0.24** (**0.07**-**0.78**)
FEF_25–75_ (% of predicted value)	69.3 ± 25.6	89.3 ± 21.9	**0.048**	**0.31** (**0.11**-**0.84**)

*SD* standard deviation, *HRCT* high-resolution computed tomography, *FVC* forced vital capacity; FEV_1_: forced expiratory volume in 1 second; FEF_25–75_: forced expiratory flow from 25 % to 75 % of vital capacity. *χ^2^ or Fisher’s exact test (categorical variables); Wilcoxon’s rank-sum test (continuous variables). Significant differences are reported in bold. ** ORs from logistic regression models for an increment equal to 1 SD, calculated in patients with RP alone, after adjustment for age at diagnosis

## Discussion

It has been demonstrated that RP early in life is a major risk factor for BE [9], but only some children actually develop BE after the first episodes of pneumonia. The early identification of the patients at the highest risk of BE could allow a diagnosis to be made when the bronchial wall lesions are still mild, thus favouring the implementation of appropriate preventive and therapeutic measures, and a better final prognosis.

More than 90 % of the children involved in this study had underlying clinical conditions that are usually considered to be predisposing factors for the development of RP. These were frequently common and only relatively severe diseases such as chronic rhinosinusitis with post-nasal drip and recurrent wheezing, whereas severe chronic underlying diseases were rare. The presence of chronic rhinosinusitis, that is often difficult to diagnose, is not surprising because sinus infections are very common childhood and may give rise to a reservoir of bacterial pathogens that chronically enter the lower airways [15, 18]. Furthermore, recurrent wheezing may be associated with the spread of viral pathogens from the upper to the lower respiratory tract [19], thus contributing to further chronic inflammation [20, 21]. It is therefore clearly important to evaluate the history of children with RP but, as rhinosinusitis and wheezing are extremely common, their presence per se does not immediately suggest the need for HRCT unless they are associated with other, more severe conditions that are known to increase the risk of BE. On the other hand, after this study we have also decided to modify the protocol active in our Unit and since 2014 we avoided to perform HRCT in a child only for RP if there are no other clinical indications to perform it.

The findings of this study seem to indicate that in a relevant number of children with RP, independently from its origin, non-CF BE is accompanied by reduction in FEV_1_ and FEF_25–75_ values, and increased responsiveness to bronchodilators. It is possible that the fact that the patients with non-CF BE were somewhat older than those without BE may have influenced the lung function results and the difference between the two group of subjects. Moreover, the absence of significant differences in responsiveness to bronchodilators can be explained by the limited number of the study patients. However, as inflammation characterises the pathogenesis of non-CF BE, it can be assumed that allergic IgE-mediated inflammation may play a concomitant role in damaging airway tissue. Horváth et al. have shown that the level of exhaled nitric oxide (FeNO), a non-invasive marker of eosinophilic inflammation, is significantly increased in adult patients with non-CF BE [22], and Narang et al. found that median levels of FeNO are higher in children with non-CF BE than in those with CF BE [23].

As we found that BE-related lesions were mainly localised, and that the non-CF BE pattern was predominantly mild, it can be hypothesised that these changes do not significantly affect spirometric values and that children with non-CF BE have pre-existing impaired lung function due to airflow obstruction, the small size of their airways, or both. On the other hand, it is also possible that our findings could reflect localized airway narrowing where the BE is rather than generalized airway narrowing. Regamey et al. recently documented an early increase in airway smooth muscle mass in children with BE [24]. The nature of the underlying chronic inflammation in the BE group was not specified, but the airway alterations undoubtedly contributed to airway narrowing. The authors also found a linear relationship between airway smooth muscle content and bronchodilator responses [24].

These findings needs confirmation with further studies because they could have a number of practical implications. The changes associated with non-CF BE can originate early in life and so possible airway resistance and inflammation should be evaluated even in very young children with RP (particularly those without a severe chronic underlying disease). Although we studied a difficult to assess age-group and there could be some limits in the repeatability of the measures, advances in spirometry measurement techniques have made it possible to obtain spirometry in children as young as 3 years of age with an appropriate repeatibility. Moreover, recently spirometry centiles were created using the lambda, micro, sigma (LMS) method extending previously published equations down to the first years of life. This permits to have reliable data also for younger children and to adequately follow bronchial function modification during development [25, 26].

The limitations of this study include its retrospective nature, the relatively small number of subjects, and the fact that variables such as FeNO and airway resistance measurements were not recorded. Nevertheless, the finding that bronchial obstruction seems to be associated with an increased risk of developing non-CF BE development is interesting, and suggests that further studies should be carried in order to confirm the value of spirometry as a means of raising the suspicion of non-CF BE in children with RP.

## Conclusions

Reduced FEV_1_ and FEF_25–75_ values seem to be an early marker of an increased risk of developing non-CF BE in children with RP.

## Abbreviations

CPAP: continuous positive airway pressure
FEF_25–75_: forced expiratory flow from 25 % to 75 % of vital capacity
FEV_1_: forced expiratory volume in 1 second
FVC: forced vital capacity
HRCT: high-resolution computed tomography
non-CF BE: non-cystic fibrosis bronchiectasis
RP: recurrent pneumonia
SD: standard deviation
